# Supplementary material for: Causal Relationship between Aging and Anorexia Nervosa: A White-Matter-Microstructure-Mediated Mendelian Randomization Analysis
Source: Biomedicines. 2024 Aug 16;12(8):1874. doi: 10.3390/biomedicines12081874 (PMC11351342; doi:10.3390/biomedicines12081874)
Supplement: Supplementary file 1 [file biomedicines-12-01874-s001.zip › Supplementary Figure/Figure S8/Leaveoneout plot of MR analysis for TL to ubm-b-1936.pdf]

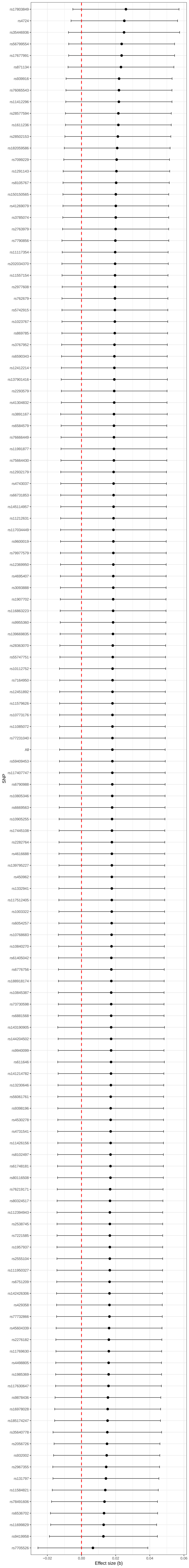

rs17803849
rs4724
rs35446936
rs56799554
rs17677991
rs871134
rs939916
rs76065543
rs11412296
rs28577594
rs1611236
rs28502153
rs182059586
rs7099229
rs1291143
rs8105767
rs150150565
rs41269079
rs3785074
rs2763979
rs7790856
rs11117354
rs202034370
rs11557154
rs2977608
rs762679
rs5742915
rs1023767
rs869785
rs3767952
rs6590343
rs12412214
rs137901416
rs2293579
rs41304832
rs3891167
rs6584579
rs76666449
rs11991877
rs75664430
rs12932179
rs4743037
rs66731853
rs145114957
rs11212631
rs117034449
rs9600019
rs79977579
rs12369950
rs4695407
rs3093888
rs1907702
rs116863223
rs9955360
rs139669835
rs28363070
rs55747751
rs10112752
rs7164950
rs12451892
rs11579626
rs10773176
rs11085072
rs77231040
All
rs59409453
rs117407747
rs6790988
rs10805346
rs6669563
rs10905255
rs17445108
rs2282764
rs4616688
rs139795227
rs450962
rs1332941
rs117512405
rs1003322
rs6054257
rs10768683
rs10840270
rs61405042
rs6776756
rs188918174
rs10845387
rs73730598
rs6881568
rs143190905
rs144204502
rs9940099
rs611646
rs141214782
rs13230646
rs56061761
rs9398196
rs4530278
rs4731541
rs11426156
rs8102497
rs61748181
rs80116508
rs76219171
rs80324517
rs112394943
rs2538745
rs7221585
rs1957937
rs2555104
rs111950327
rs6751209
rs142426306
rs429358
rs77732866
rs45604339
rs2276182
rs11769630
rs4498805
rs1985369
rs117630647
rs9878436
rs16978028
rs185174247
rs35640778
rs2056726
rs932002
rs2967355
rs131797
rs11584821
rs78491606
rs6536702
rs11699829
rs9419958
rs7705526
SNP
−0.02
0.00
0.02
0.04
0.06
Effect size (b)
